# Supplementary material for: Deficiency in coatomer complex I causes aberrant activation of STING signalling
Source: Nat Commun. 2022 Apr 28;13:2321. doi: 10.1038/s41467-022-29946-6 (PMC9051092; doi:10.1038/s41467-022-29946-6)
Supplement: Supplementary file 1 — Supplementary Information [file 41467_2022_29946_MOESM1_ESM.pdf]

Supplementary Information

Deficiency in coatomer complex I causes aberrant activation of STING signalling

Steiner et al.

Corresponding author: Seth L. Masters (masters@wehi.edu.au)

This PDF file includes:

Supplementary Figures 1-10
Supplementary Tables 1-3

Other Supplementary Materials:

Supplementary Data 1 (Excel file)

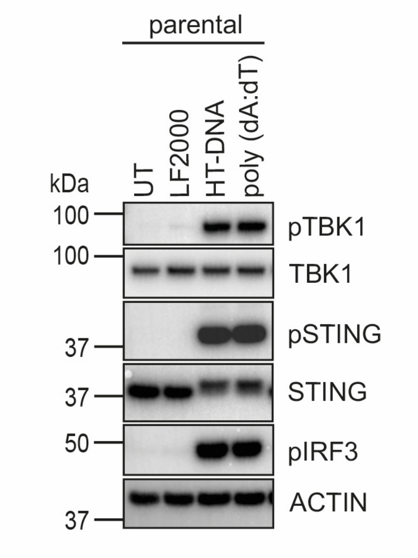

Supplementary Figure 1 | The cGAS/STING pathway is functional in parental HeLa cells used in this study.

Parental HeLa cells were lipofectamine (LF2000)-transfected with cGAS activators HT-DNA (2 µg/ml) and poly (dA:dT) (1 µg/ml) for 90 min and immunoblotted for detection of cGAS/STING signalling pathway proteins, representative result of n=3. Source data are provided at the end of the Supplementary Information file.

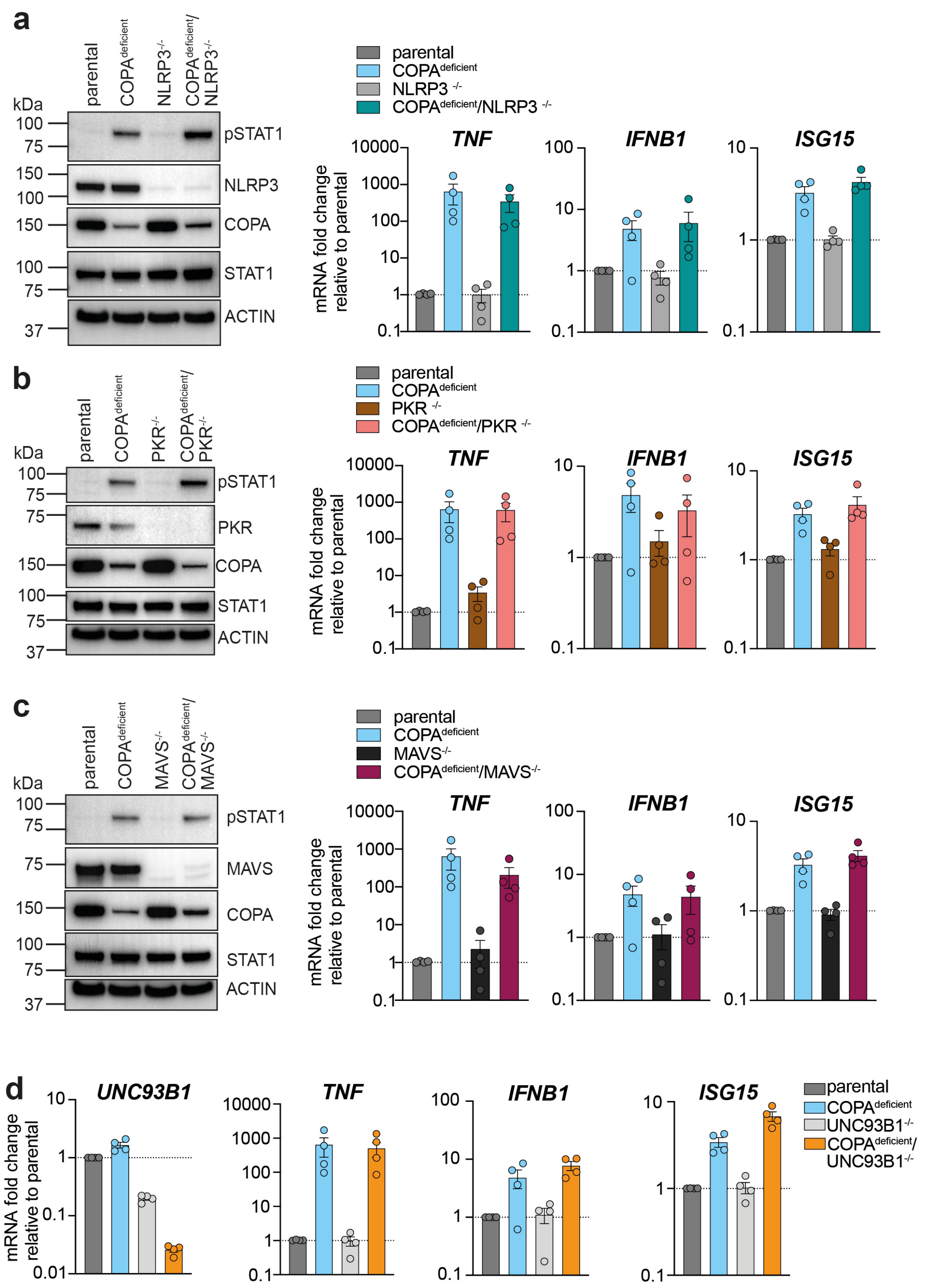

Supplementary Figure 2 | Inflammatory signalling associated with COPA-deficiency in not mediated through NLRP3-, PKR-, MAVS-, UNC93B1-dependent pathways.

The effect of CRISPR/Cas9-mediated genetic deletion of NLRP3 (**a**), PKR (**b**), MAVS (**c**), UNC93B1 (**d**) in COPA^{deficient} THP-1 cells was assessed by immunoblot analysis of phosphorylated STAT1 (pSTAT1) and transcription analysis of *TNF*, *IFNB1*, *ISG15 and UNC93B1* by qRT-PCR. Data are presented as mean ± SEM from 4 independent experiments. Source data are provided at the end of the Supplementary Information file.

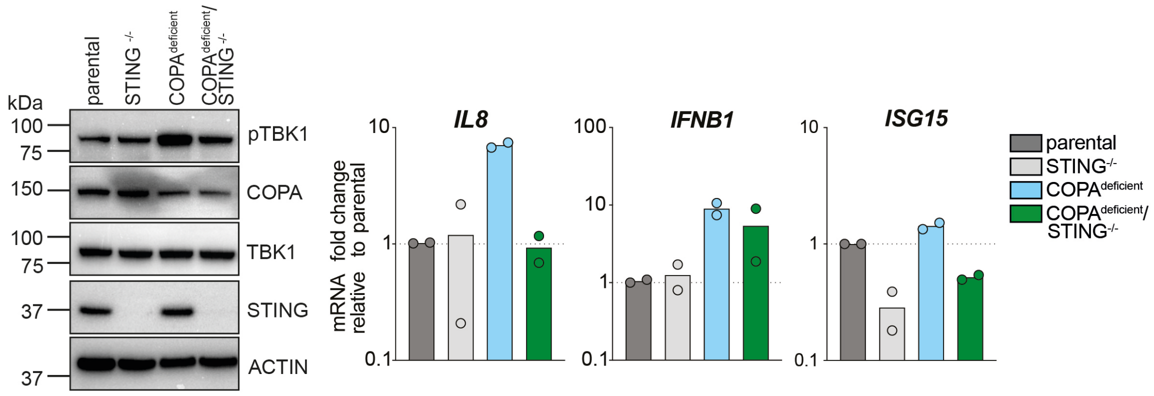

Supplementary Figure 3 | Inflammation in COPA^{deficient} HeLa cells is STING-dependent.

CRISPR/Cas9 gene editing was performed to genetically delete STING and generate COPA^{deficient} HeLa cells. Protein expression levels of COPA, STING and phosphorylated TBK1 (pTBK1) were assessed by immunoblot analysis of unstimulated cells. A representative experiment of n=2 is shown. Inflammatory pathway activation was also investigated by qRT-PCR analysis of proinflammatory gene transcription at baseline. Data are presented as mean from n=2 independent experiments showing individual data points. Source data are provided at the end of the Supplementary Information file.

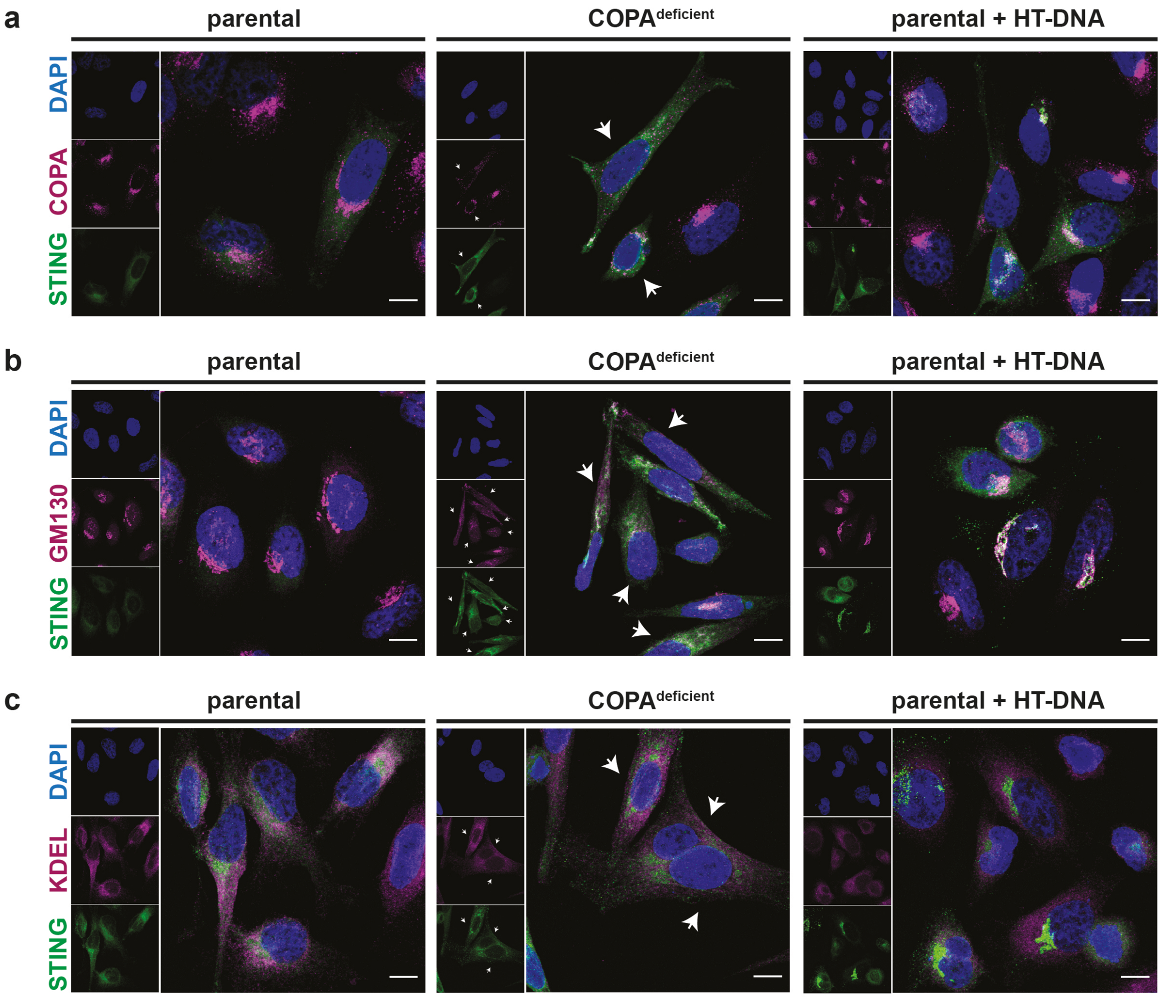

Supplementary Figure 4 | STING-GFP co-localizes with the dispersed Golgi in COPA^{deficient} HeLa cells.

Parental and COPA^{deficient} HeLa cells stably reconstituted with STING-GFP were Dox-treated for 72 hrs, fixed and stained for COPA **(a)**, GM130 **(b)** or KDEL **(c)**. Localization of activated STING-GFP (green) in parental HeLa cells transfected with HT-DNA (2 µg/ml, 2 hrs) results in STING-GFP accumulation at the Golgi (GM130), which is shown as positive control. Representative images, n=1, scale bar 10 µm, white arrows indicate COPA^{deficient} cells.

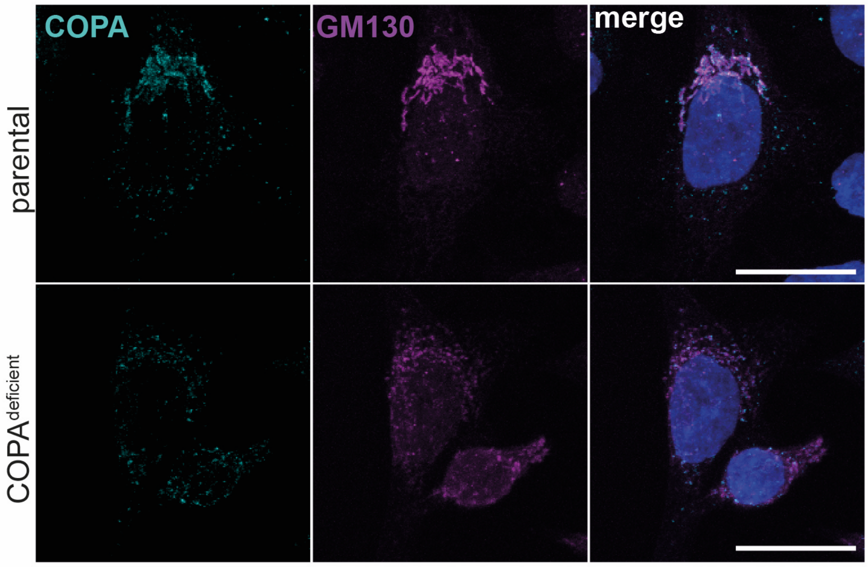

Supplementary Figure 5 | Deletion of COPA results in Golgi dispersal.

Immunofluorescence analysis of parental and COPA^{deficient} HeLa cells after 72 hrs of Dox treatment stained for COPA (cyan), cis-Golgi marker GM130 (magenta) and DAPI (blue). Pictures are representatives of n=2 independent experiments. Scale bar: 20 μm.

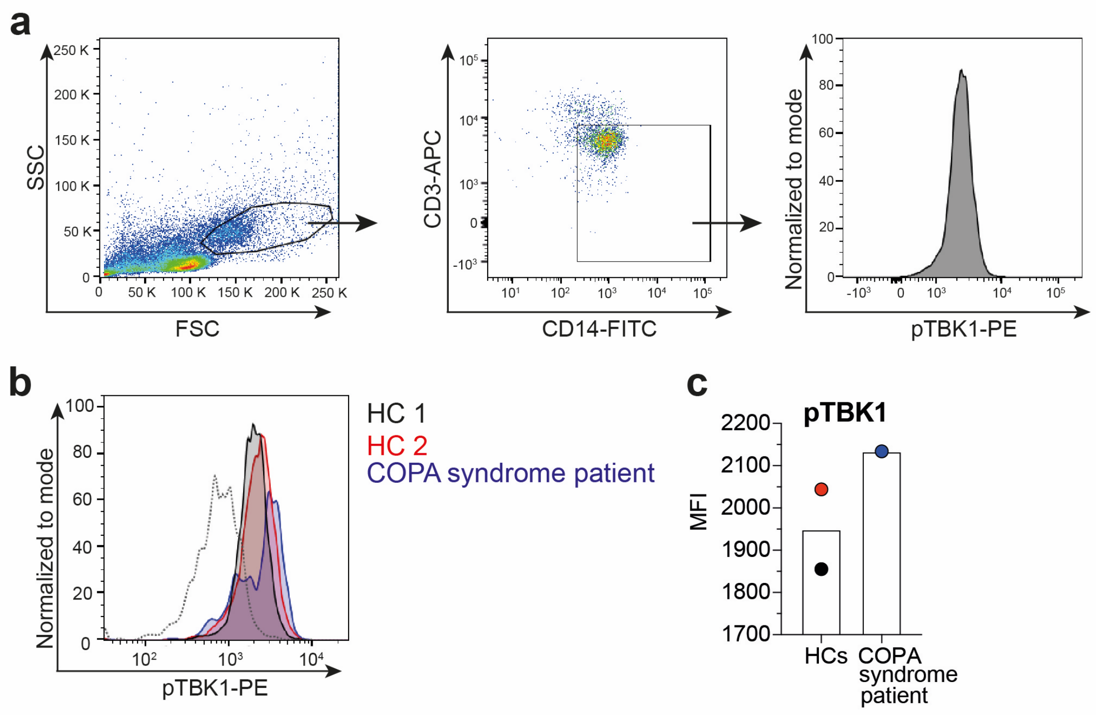

Supplementary Figure 6 | Phosphorylated TBK1 is elevated in monocytes isolated from COPA syndrome patients.

a) Flow cytometry pseudocolor plots exemplifying the gating strategy used to analyse PBMC samples. The monocyte population was firstly identified based on cell size (forward scatter, FSC) and granularity (side scatter, SSC) and subsequently confirmed by gating for the CD14-FITC positive and CD3-APC negative subpopulation. The phosphorylated TBK1 (pTBK1-PE) signal was analysed using histograms normalized to mode to allow overlayed comparison between different samples. **b)** Flow cytometry analysis comparing the pTBK1-PE in the monocytic subpopulation isolated from COPA syndrome patient PBMCs (blue) and 2 healthy individuals (HCs, black, red) without further stimulation. Histogram shows the pTBK1 signal from this experiment, dotted line indicates the isotype control. **c)** Column graph quantifies data shown in b) as geometric mean fluorescence intensity (MFI) of the same experiment using 2 independent HCs (line at mean), n=1.

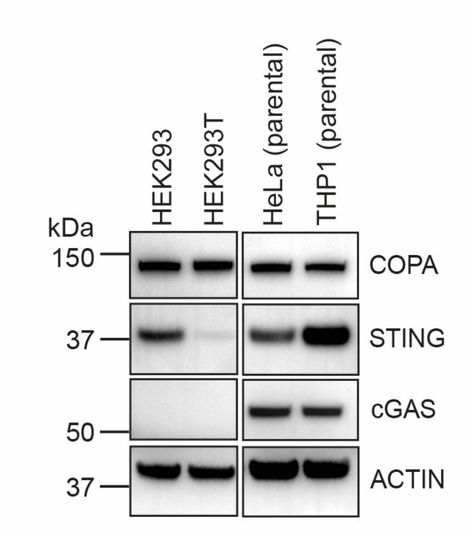

Supplementary Figure 7 | Endogenous cGAS/STING expression in cell lines used throughout this study.

Immunoblot analysis of endogenous STING and cGAS expression levels in HEK293, HEK293T, parental HeLa and THP-1 cell lines used in this study. A representative western blot of n=2 independent experiments is shown. Source data are provided at the end of the Supplementary Information file.

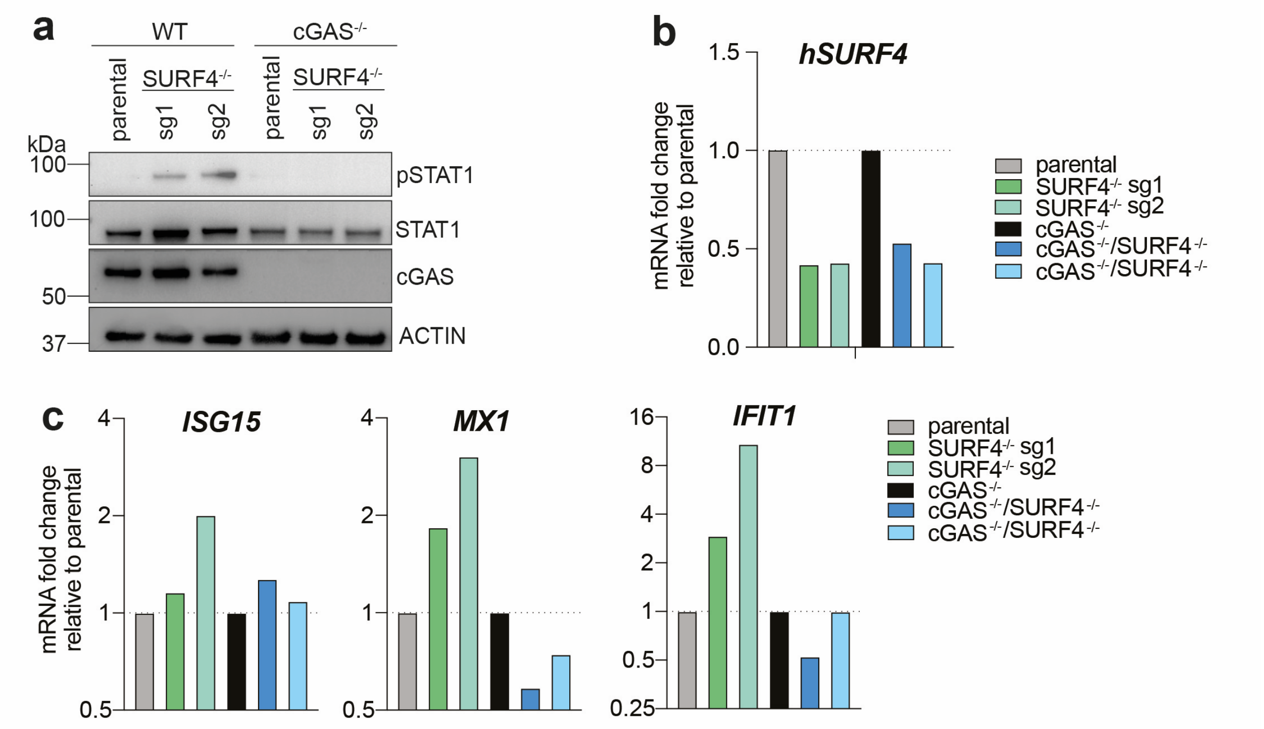

Supplementary Figure 8 | Genetic deletion of adapter protein SURF4 induces inflammatory signalling in a cGAS-dependent manner.

a) Immunoblot analysis of inflammatory signalling in THP-1 parental (WT) and monoclonal cGAS$^{-/-}$ THP-1 cells following genetic deletion of SURF4 using 2 different sgRNAs (sg1 and sg2), n=1. **b)** qRT-PCR analysis of SURF4 transcription levels of cell lines used in a). **c)** qRT-PCR analysis of ISG transcription levels in THP-1 cell lines used in a) and b). **b)** and **c)** Data are shown as mean from 2 technical replicates, n=1. Source data for a) are provided at the end of the Supplementary Information file.

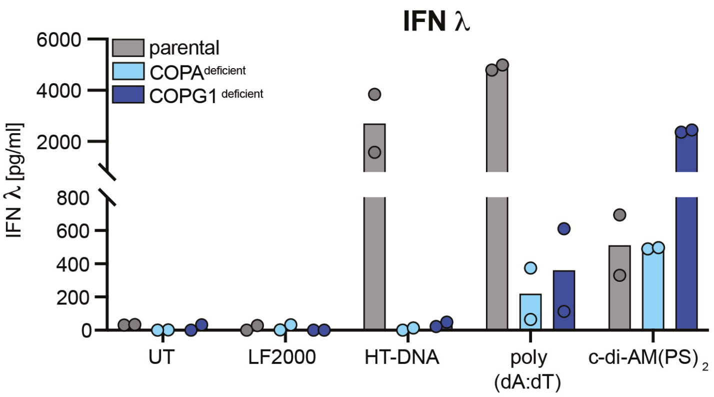

Supplementary Figure 9 | cGAS stimulation is impaired in COPA- and COPG1-deficient THP-1 cells.

THP-1 cells deficient for COPA and COPG1 were stimulated with HT-DNA (2 µg/ml), poly (dA:dT) (1 µg/ml) and c-di-AM(PS)$_2$ (20 µM). After 24 hrs stimulation supernatants were analysed for IFNλ by ELISA. Data are shown as mean from n=2 independent experiments showing individual data points.

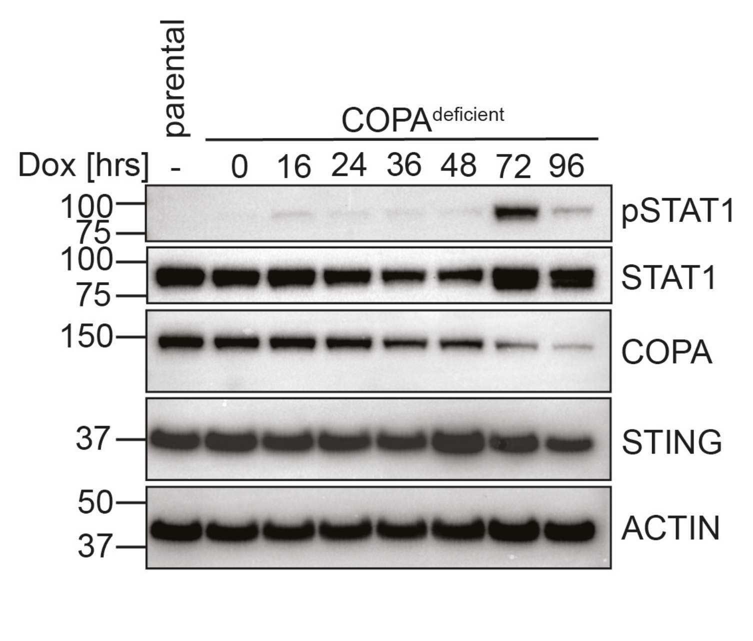

Supplementary Figure 10 | Loss of COPA does not alter cellular expression levels of STING.

Western blot analysis of STING expression levels in THP-1 cells during 96 hrs Dox treatment when COPA protein levels gradually decline. A representative result of n=3 independent experiments is shown. Source data are provided at the end of the Supplementary Information file.

Supplementary Table 1 | Single guide (sg) RNAs for CRISPR/Cas9-mediated gene editing targeting human genes.

Target gene	sgRNA sequence
COPA (sgRNA 1)	TAGATTGCCAGTTCCACACT
COPA (sgRNA 2)	AATTCGAGACCAAGAGCGCG
COPA (sgRNA 3)	ACATCCGATTCCACCGCACC
STING	AGAGCACACTCTCCGGTACC
NLRP3	TCCCGCTGGACCATCCTCGGCATG
MAVS	AGTACTTCATTGCGGCACTG
PKR	TAATACATACCGTCAGAAGC
ARCN1 (COPD)	ATACCGGGAGAATGTTAACT
COPE	ACTACCTCGCCCACGAGAGT
COPG1	TGGTCAAGTAGCACATCCGA
COPG2	GGAAAAGAAGATGTATACCG
SURF4 (sgRNA 1)	TCAGACAGAGGCGCGCCACG
SURF4 (sgRNA 2)	AGTCGCGCTGCTCGCTCCAC
UNC93B1	GGGCGTGCTCAAGAACGTGC

Supplementary Table 2 | QuikChange mutagenesis primer used in this study (F; forward, R; reverse).

Target gene and mutation	Primer sequence (5’→3’)
hCOPA_p.R233H_F	AGTGAAGATCTGGCACATGAATGAATCAAAGGC
hCOPA_p.R233H_R	GCCTTTGATTCATTCATGTGCCAGATCTTCACT
hCOPA_p.E241K_F	TCAAAGGCATGGAAGGTTGATACCTGC
hCOPA_p.E241K_R	GCAGGTATCAACCTTCCATGCCTTTGA

Supplementary Table 3 | Human primer sequences for quantitative Real-Time (qRT)-PCR for detection with SYBR Green (F; forward, R; reverse).

Target gene	F/R	Sequence (5' → 3')
ACTIN	F	GCGAGAAGATGACCCAGATC
	R	CCAGTGGTACGGCCAGAGG
COPA	F	ACTGGCAATCTAGAACCTGTG
	R	GACCAGAAATATCCCAAACGC
TNF	F	TCTCTCAGCTCCACGCCATT
	R	CCCAGGCAGTCAGATCATCTTC
IFNA1	F	GCCTCGCCCTTTGCTTTACT
	R	CTGTGGGTCTCAGGGAGATCA
IFNB1	F	TGTCGCCTACTACCTGTTGTGC
	R	AACTGCAACCTTTCGAAGCC
ISG15	F	TCCTGGTGAGGAATAACAAGGG
	R	GTCAGCCAGAACAGGTCGTC
IFIT1	F	ATCCACAAGACAGAATAGCCAG
	R	CCAGACTATCCTTGACCTGATG
MX1	F	GTTTCCGAAGTGGACATCGCA
	R	CTGCACAGGTTGTTCTCAGC
USP18	F	CCTGAGGCAAATCTGTCAGTC
	R	CGAACACCTGAATCAAGGAGTTA
IL6	F	TCAATATTAGAGTCTCAACCCCCA
	R	GAAGGCGCTTGTGGAGAAGG
IL8	F	CTGGCCGTGGCTCTCTTG
	R	CCTTGGCAAAACTGCACCTT
COPG2	F	CCTAAGCCAGCCTTGAGATATG
	R	GTCCAGATTGCAGGCAGTAA
SURF4	F	GGTCTTGCTGGTTCTGATGT
	R	CCCACGATGTTCTGGACAATA
IL1B	F	AATCTGTACCTGTCCTGCGTGTT
	R	TGGGTAATTTTTGGGATCTACACTCT
UNC93B1	F	TCAAGAACGTGCTGGCTGCC
	R	GGCGATGGGAGTCACGTTGAT
