# Supplementary material for: Deficiency in coatomer complex I causes aberrant activation of STING signalling
Source: Nat Commun. 2022 Apr 28;13:2321. doi: 10.1038/s41467-022-29946-6 (PMC9051092; doi:10.1038/s41467-022-29946-6)
Supplement: Supplementary file 3 — Description of Additional Supplementary Files [file 41467_2022_29946_MOESM3_ESM.docx]

**Description of Additional Supplementary Files**

**Supplementary Data 1 | Full list of potential STING-interacting proteins identified by mass spectrometry-based quantitative proteomics.**

Proteins identified by mass spectrometry after transient overexpression of mCitSTING and pulldown in HEK293T cells relative to empty vector (EV) control. Differential expression analysis was performed using limma. Data have been deposited to the ProteomeXchange Consortium via the PRIDE partner repository with the dataset identifier PXD023135. Cellular localisation of identified potential STINGinteracting proteins to ER or Golgi compartments was determined using the Gene Ontology database (https://www.ebi.ac.uk/QuickGO/, particularly cellular component terms). The identified ER- and Golgi-resident proteins were allocated accordingly to two separate tabs in the excel sheet containing Supplementary Table 1. Presence of C-terminal lysine (K), arginine (R) and histidine (H) residues of identified Golgi- and ER-resident proteins was highlighted (bold) to indicate potential COPI complex binding ability. However, only clearly identified COPI-binding motifs (KKxx, RKxx, KxKxx, KxRxx, KDEL/RDEL/HDEL) were underlined and stated. Identified proteins contributing to COPI/COPII complex formation were highlighted in green and previously identified STING interactors are highlighted in grey.
