# Supplementary material for: Deficiency in coatomer complex I causes aberrant activation of STING signalling
Source: Nat Commun. 2022 Apr 28;13:2321. doi: 10.1038/s41467-022-29946-6 (PMC9051092; doi:10.1038/s41467-022-29946-6)
Supplement: Supplementary file 5 — Reporting Summary [file 41467_2022_29946_MOESM5_ESM.pdf]

nature research

Corresponding author(s): Masters and Davidson

Last updated by author(s): Sept 20, 2021

Reporting Summary

Nature Research wishes to improve the reproducibility of the work that we publish. This form provides structure for consistency and transparency in reporting. For further information on Nature Research policies, see our Editorial Policies and the Editorial Policy Checklist.

Statistics

For all statistical analyses, confirm that the following items are present in the figure legend, table legend, main text, or Methods section.

n/a	Confirmed	
☐	☒	The exact sample size (n) for each experimental group/condition, given as a discrete number and unit of measurement
☐	☒	A statement on whether measurements were taken from distinct samples or whether the same sample was measured repeatedly
☐	☒	The statistical test(s) used AND whether they are one- or two-sided<br>*Only common tests should be described solely by name; describe more complex techniques in the Methods section.*
☐	☒	A description of all covariates tested
☐	☒	A description of any assumptions or corrections, such as tests of normality and adjustment for multiple comparisons
☐	☒	A full description of the statistical parameters including central tendency (e.g. means) or other basic estimates (e.g. regression coefficient) AND variation (e.g. standard deviation) or associated estimates of uncertainty (e.g. confidence intervals)
☒	☐	For null hypothesis testing, the test statistic (e.g. F, t, r) with confidence intervals, effect sizes, degrees of freedom and P value noted<br>*Give P values as exact values whenever suitable.*
☒	☐	For Bayesian analysis, information on the choice of priors and Markov chain Monte Carlo settings
☒	☐	For hierarchical and complex designs, identification of the appropriate level for tests and full reporting of outcomes
☒	☐	Estimates of effect sizes (e.g. Cohen's d, Pearson's r), indicating how they were calculated

Our web collection on statistics for biologists contains articles on many of the points above.

Software and code

Policy information about availability of computer code

Data collection	Impact II UHR-QqTOF mass spectrometer (Bruker), nano-flow HPLC (M-class, Waters), ViiA 7 Real-time PCR system (Thermo Fisher Scientific), Leica SP8 Resonant Scanning Confocal (Leica Microsystems) using Leica Application Suite X (LAS X) 3.5.7.23225 software, Zeiss LSM 880 NLO fast Airyscan Confocal using ZEN 2.3 SP1 (Zeiss), DeltaVision OMX-SR system (GE Healthcare) using softWoRx 7.0.0, FACSCanto flow cytometer (BD Biosciences).
Data analysis	MaxQuant (version 1.6.6.0), Fiji (version 2.1.0), FlowJo (version 10.5), GraphPad Prism (version 8)

For manuscripts utilizing custom algorithms or software that are central to the research but not yet described in published literature, software must be made available to editors and reviewers. We strongly encourage code deposition in a community repository (e.g. GitHub). See the Nature Research guidelines for submitting code & software for further information.

Data

Policy information about availability of data

All manuscripts must include a data availability statement. This statement should provide the following information, where applicable:

- Accession codes, unique identifiers, or web links for publicly available datasets
- A list of figures that have associated raw data
- A description of any restrictions on data availability

Data supporting the findings reported in this study are presented within the results section and the supplementary files. All graphs show individual data points pooled from replicate experiments. Raw data are available upon request from the corresponding author. The mass spectrometry proteomics data have been deposited to the ProteomeXchange Consortium via the PRIDE partner repository (http://proteomecentral.proteomexchange.org/cgi/GetDataset) with the dataset identifier PXD023135. Localization of identified STING-interacting proteins to ER or Golgi compartments in Supplementary Table 1 was determined using the Gene Ontology database (https://www.ebi.ac.uk/QuickGO/, particularly cellular component terms). Other databases used include Uniprot (https://www.uniprot.org/) and

LFQAnalyst (https://bioinformatics.erc.monash.edu/apps/LFQ-Analyst/). Source data are available with this paper for Figures 1a, 1d, 2b, 2d, 3c-e, 4a, 5c, 5d, 6a, 6d and supplementary figures 1, 2a-c, 3, 7, 8a and 10.

Field-specific reporting

Please select the one below that is the best fit for your research. If you are not sure, read the appropriate sections before making your selection.

- [x] Life sciences
- [ ] Behavioural & social sciences
- [ ] Ecological, evolutionary & environmental sciences

For a reference copy of the document with all sections, see nature.com/documents/nr-reporting-summary-flat.pdf

Life sciences study design

All studies must disclose on these points even when the disclosure is negative.

Sample size	Patient data utilized all samples that were available to us (n=1). For in vitro experiments using cell lines, no sample size calculations were performed. For the majority of experiments, three biologically independent repeats were performed based on the number of replicates used in other studies using similar methods (e.g. PMID: 35148201).
Data exclusions	There were no data exclusions unless a technical error was detected and flagged during the experiment.
Replication	The patient study (n=1) has not yet been replicated due to limitations of sample availability. The majority of in vitro studies using cell lines were replicated independently as stated in the respective figure legends, each time yielding similar results. For most experiments, three independent replicates were performed, with exceptions of experiments shown in Fig. 4a (n=2), 4c (n=2), 4d (n=2), 5 b-c (n=2), 6 a-c (n=2), Suppl. Fig. 3 (n=2), Suppl. Fig. 5 (n=2), Suppl. Fig. 7 (n=2), Suppl. Fig. 9 (n=2). Preliminary results are shown in Suppl. Fig. 4 (n=1) and Suppl. Fig. 8 (n=1).
Randomization	Randomization was not required for the in vitro studies using immortalized cell line models, since these are assumed to be identical when seeded into multiple wells for experimental analysis. For the patient study, the experimenter was blinded during selection of the healthy control samples.
Blinding	The patient study was conducted in a blinded fashion. For in vitro studies using cell line models, sample collection or data analysis were not blinded, since these experiments were not susceptible to bias.

Reporting for specific materials, systems and methods

We require information from authors about some types of materials, experimental systems and methods used in many studies. Here, indicate whether each material, system or method listed is relevant to your study. If you are not sure if a list item applies to your research, read the appropriate section before selecting a response.

Materials & experimental systems

n/a	Involved in the study	
☐	☒	Antibodies
☐	☒	Eukaryotic cell lines
☒	☐	Palaeontology and archaeology
☒	☐	Animals and other organisms
☐	☒	Human research participants
☒	☐	Clinical data
☒	☐	Dual use research of concern

Methods

n/a	Involved in the study	
☒	☐	ChIP-seq
☐	☒	Flow cytometry
☒	☐	MRI-based neuroimaging

Antibodies

Antibodies used	anti-COPA (Santa Cruz Biotechnology, clone H-3, sc-398099), anti-COPD (Santa Cruz Biotechnology, clone E-12, sc-515549), anti-COPE (Santa Cruz Biotechnology, clone A-4, sc-133195), anti-phospho-STAT1 Tyr701 (Cell Signaling Technology, clone 58D6, #9167), anti-phospho-TBK1/NAK Ser172 (Cell Signaling Technology, clone D52C2, #5483), anti-phospho-IRF3 Ser386 (Abcam, ab76493, EPR2346), anti-STING (Cell Signaling Technology, clone D2P2F, #13647), anti-STAT1 (Cell Signaling Technology, clone D1K9Y, #14994), anti-TBK1/NAK (Cell Signaling Technology, #3013), anti-cGAS (D1D3G, Cell Signaling Technology #15102), anti-GFP (Life Technologies, #A11122), anti-Myc-Tag (Cell Signaling Technology, clone 9B11, #2276), anti-COPG (A-10, sc-393977, Santa Cruz Biotechnology), anti-iNOS/NOS type II (BD Transduction Laboratories, #610329), anti-NLRP3 (AdipoGen Life Sciences, Cryo-2, AG-20B-0014-C100, dilution 1:1000), anti-PKR (Santa Cruz Biotechnology, clone B-10, sc-6282, dilution 1:1000), anti-Cardif (AdipoGen Life Sciences, Adri-1, AG-20B-0004-C100, dilution 1:500), anti-Actin-HRP (Santa Cruz Biotechnology, clone C4, sc-47778), anti-COPG (Cat. No. 12393-I-AP, ProteinTech), anti-COPD (Cat. No. GTX630562, clone GT1318, GeneTex), anti-GM130-AF647 (ab195303, EP892Y, Abcam), anti-KDEL (clone 10C3, ab12223, Abcam), anti-KDEL-AF568 (ab203421, EPR12668, Abcam).
Validation	-COPA antibody (sc-398099): mouse monoclonal antibody raised against amino acids 934-1233 mapping at the C-terminus of human COPA. Recommended for detection of mouse, human and rat COPA, tested in cell

lysates of several cell lines. Suitable for WB, IP, IHC, IF. Validated using CRISPR/Cas9-mediated deletion and overexpression of COPA within the manuscript (Fig. 1a, 2f, 3c-e) and previously in siRNA-transfected normal human dermal fibroblasts (McCormick et al. 2018 mBio). Used in other publications: Kuliyev et al. 2018 J. Neurosci., Baron et al. 2019 Mol. Genet., Guo et al. 2021 PLoS Pathog.
-COPD antibody (sc-515549): mouse monoclonal antibody raised against amino acids 254-511 mapping at the C-terminus of COPD. Recommended for detection of mouse, human and rat COPD. Suitable for WB, IF, IP, IHC. Tested in cell lysates of several cell lines. Used in previously published studies: Barbera et al. 2019 Cell Commun. Signal. Further validated using CRISPR/Cas9-mediated deletion of COPD in the manuscript (Fig. 5d).
-COPE antibody (sc-133195): mouse monoclonal antibody raised against amino acids 111-190 of COPE of human origin. Recommended for detection of mouse, human and rat COPE, tested in cell lysates of several cell lines. Suitable for WB, IF, IP, IHC. Further validated using CRISPR/Cas9-mediated deletion of COPE in the manuscript (Fig. 5d).
- anti-phospho-STAT1 Tyr701 (#9167): reactivity against human and mouse, recommended for WB, IP, IHC, IF, flow cytometry. Previously used in published studies including Sun et al. 2022 Front Microbiol, Chikhalya et al. 2021 MBio. Further validated using suitable controls in this manuscript (Fig. 1a, 2b, 2d).
- anti-phospho-TBK1/NAK Ser172 (#5483): reactivity against human and mouse, recommended for WB, IP, IF, flow cytometry. Possible cross-reactivity with phospho-IKK. Rat, monkey, xenopus, bovine, dog are predicted to react based on sequence homology. Used in many publications including Chen et al. 2022 Bioact Mater., Kim et al. 2021 PLoS One. Further validated using appropriate positive and negative controls in this manuscript (Fig. 3c-e).
- anti-phospho-IRF3 Ser386 (ab76493): reactivity against human protein, recommended for WB and dot plot. Previously used in many published studies. Further validated in this manuscript using appropriate positive and negative controls (Fig. 3c-e).
- anti-STING (#13647): reactivity against human and mouse, suitable for WB, IP, IHC. Used in numerous scientific publications, further validated in this manuscript using CRISPR/Cas9-mediated STING deletion (Fig. 2b) and overexpression experiments (3c-e).
-anti-STAT1 (#14994): reactivity against human, mouse, rat, monkey. Suitable for WB, IP, IHC, IF, flow cytometry, ChIP. Knockout-validated in A549 cells. Cross-reactivity with an unidentified protein of 150 kDa possible.
- anti-TBK1/NAK (#3013): reactivity against human, mouse, rat, monkey. Suitable for WB, IP. Used in several published studies.
- anti-cGAS (#15102): reactivity against human protein. Suitable for WB. Validated by overexpression of myc-tagged full-length human cGAS in HEK293T cells, able to detect endogenous protein in several cell lines, product has been cited in several published studies. Further validated in this manuscript using the monoclonal cGAS knockout THP-1 cell line (Fig. 4a).
- anti-GFP (#A11122): has previously been used for several applications including WB, IHC, IF, Flow cytometry, EILSA. Validated by WB analysis of several GFP-tagged target proteins in cell lysates of transiently transfected HEK293E cells. Cross-reactivity with YFP.
- anti-Myc-Tag (#2276): reactivity against myc-tagged proteins regardless of N-or C-terminal tag location. Suitable for WB, IP, IHC, IF, flow cytometry, ChIP. The antibody shows a weak cross-reactivity with an unknown protein of 90 kDa.
- anti-COPG (sc-393977): raised against amino acids 849-874 at the C-terminus of mouse COPG with reactivity against human, mouse, rat COPG protein. Suitable for WB, IP, IF, IHC, ELISA. Used in previously published studies and further validated in this manuscript using CRISPR/Cas9-mediated deletion of COPG (Fig. 5d).
- anti-iNOS/NOS type II (#610329): reactivity against mouse and human. Suitable for WB, IHC, IP. Used in several publications including Paterniti et al. 2017 PLoS ONE and Lindsay et al. 2016 Stem Cell Reports.
- anti-NLRP3 (AG-20B-0014-C100): reactivity against human and mouse. Suitable for WB, IHC, IP, ChIP. Validated in murine macrophages as per product data sheet and further validated in using CRISPR/Cas9-mediated deletion of NLRP3 in this manuscript (Supplementary Fig. 2a).
-anti-PKR (sc-6282): reactivity against mouse, rat, human, suitable for WB, IP, IF, IHC. Used in several published studies and validated using CRISPR/Cas9-mediated deletion of PKR in this manuscript (Supplementary Fig. 2b).
-anti-MAVS/anti-Cardif antibody (AG-20B-0004-C100): reactive against human, suitable for WB, IP and IHC.
- anti-Actin-HRP (sc-47778): reactivity against human, mouse, rat, avian, bovine, canine, porcine, rabbit. Suitable for WB, IP, IF, IHC applications. Used in multiple published research articles including Zou et al. 2021 Neural. Regen. Res.
-anti-COPG (12393-I-AP): reactivity against human, mouse, rat. Suitable for WB, IP, IHC, IF, ELISA. Previously used in a published study by Mukai et al 2021 Nat Commun. Further validated in this study using CRISPR/Cas9-mediated deletion of COPG (Fig. 5b).
- anti-COPD (GTX630562): Reactivity against human, mouse, rat. Suitable for WB, ICC/IF; IHC. Previously used in a published study by Mukai et al 2021 Nat Commun. Further validated in this study using CRISPR/Cas9-mediated deletion of COPD (Fig. 5b).
-anti-GM130-AF647 (ab195303): reactivity against human. Suitable for ICC/IF, flow cytometry. Staining validated in HeLa cells.
-anti-KDEL (ab12223): reactivity against human, mouse, predicted to work with S. cerevisiae, bird, plant and other mammals. Suitable for IHC, ICC/IF, WB, ELISA, IP, flow cytometry.
- anti-KDEL-AF568 (ab203421): reactivity against human, predicted to work with mouse and rat. Suitable for ICC/IF applications.

Eukaryotic cell lines

Policy information about cell lines

Cell line source(s)	HEK293 cells (CellBank Australia), HEK293T cells (ATCC), HeLa cells (Curie Institute, Paris), THP-1 cells (ATCC), clonal cGAS KO (cGAS-/-) THP-1 cell line (previously published PMID: 25425575), iBMDMs were generated inhouse (gift from Daniel Simpson (WEHI), generated following the previously published protocol (PMID: 29761386l)).
Authentication	Cell lines were not authenticated
Mycoplasma contamination	All cell lines regularly test negative for mycoplasma
Commonly misidentified lines (See ICLAC register)	No commonly misidentified lines

Human research participants

Policy information about studies involving human research participants

Population characteristics	Due to the limited availability of patients and patient samples, PBMCs from only one COPA syndrome patient (female, 10 years old) were analysed. At the time of sample collection, the patient was treated with prednisone and rituximab. A detailed clinical evaluation of this patient was previously published (PMID: 29030294), which is also referenced in the manuscript. Healthy control samples were collected from adult donors at the blood donation centre of the Istituto Giannina Gaslini and anonymized for population characteristics.
Recruitment	Regional restrictions and limited patient availability only allowed for recruitment of a single patient at the time the study was performed. Healthy control samples were recruited in a blinded fashion.
Ethics oversight	Ethical review board of Istituto Giannina Gaslini-Genova-Italy N. BIOL 6/5/04

Note that full information on the approval of the study protocol must also be provided in the manuscript.

Flow Cytometry

Plots

Confirm that:

- [x] The axis labels state the marker and fluorochrome used (e.g. CD4-FITC).
- [x] The axis scales are clearly visible. Include numbers along axes only for bottom left plot of group (a 'group' is an analysis of identical markers).
- [x] All plots are contour plots with outliers or pseudocolor plots.
- [x] A numerical value for number of cells or percentage (with statistics) is provided.

Methodology

Sample preparation	PBMCs from a COPA syndrome patient, previously reported (PMID: 29030294)), and two healthy control (HC) donors were treated withSTING inhibitor H-151 (5 µM, InvivoGen) at 37 °C for 4 hours. Cells were fixed with pre-warmed Fixation Buffer (BioLegend) at 37 °C for 15 minutes and permeabilized with pre-chilled True-Phos™ Perm Buffer (BioLegend) at -20 °C for 1 hour.
Instrument	FACSCanto (BD Biosciences)
Software	FlowJo 10.5 software
Cell population abundance	No sorting, only analysis
Gating strategy	The monocyte population was firstly identified based on cell size and granularity and subsequently confirmed by gating for CD14-positive/CD3-negative subpopulation (Supplementary Figure 6a).

- [x] Tick this box to confirm that a figure exemplifying the gating strategy is provided in the Supplementary Information.
